# Global burden of lung cancer attributable to metabolic and dietary risk factors: an overview of 3 decades and forecasted trends to 2036

**DOI:** 10.3389/fnut.2025.1534106

**Published:** 2025-03-13

**Authors:** Yuying Xiang, Yun Chen, Lingyan Lan, Shuling Chen, Qijin Shu

**Affiliations:** ^1^The First School of Clinical Medicine, Zhejiang Chinese Medical University, Hangzhou, China; ^2^The First Affiliated Hospital of Zhejiang Chinese Medical University (Zhejiang Provincial Hospital of Traditional Chinese Medicine), Hangzhou, China

**Keywords:** Global Burden of Disease, lung cancer, high fasting plasma glucose, diet low in fruits, diabetes mellitus, prediction

## Abstract

**Background:**

Lung cancer (LC) is the leading cause of cancer-related burden worldwide. Unhealthy dietary patterns and related metabolic diseases, such as diabetes mellitus (DM), represent critical global public health challenges. Nevertheless, the global burden of LC attributable to metabolic and dietary factors remains uncertain.

**Methods:**

This study aims to analyze global burden of LC attributable to metabolic and dietary risk factors, based on the Global Burden of Disease (GBD) 2021, from 1990 to 2021. Additionally, the autoregressive integrated moving average (ARIMA) model was utilized to forecast the disease burden of LC for the upcoming 15-year period.

**Results:**

High fasting plasma glucose (HFPG) and Diet low in fruits (DLF) are identified as the sole metabolic and dietary risk factors for LC, respectively, according to GBD 2021. The study findings indicate that a marked increase in the LC burden caused by HFPG, whereas the age-standardized rates (ASRs) of mortality and disability-adjusted life-years (DALYs) attributable to DLF for LC represent a general decline. At the social population index (SDI) regional level, the burden of LC attributable to DLF represents the most rapid increase in low-middle SDI regions, and while, the burden of LC attributable to DLF exhibits the most rapid decline in high-middle SDI regions. Moreover, LC burden attributable to HFPG and DLF in mortality and DALYs is higher among males than females, with sex difference being more pronounced in the elderly.

**Conclusion:**

From 1990 to 2021, the burden of LC attributed to HFPG has increased owing to the escalating exposure levels of DM, whereas the burden resulting from DLF has declined. The burden of LC attributable to HFPG and DLF exhibits distinct spatiotemporal patterns and similar gender-age patterns.

## Graphical abstract

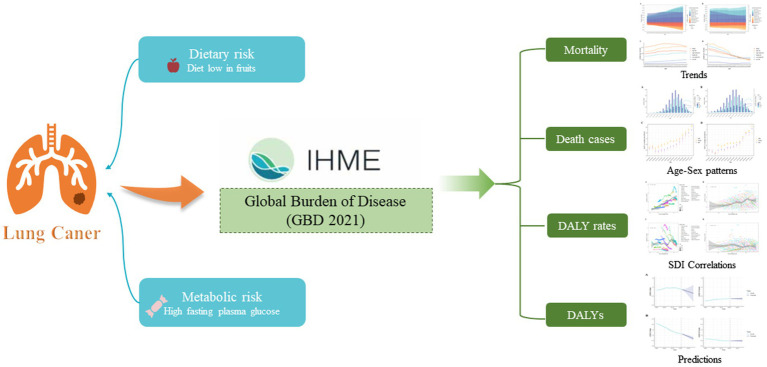

## 1 Introduction

Cancer remains a remarkable global public health concern and a prominent contributor to the overall disease burden worldwide. Lung cancer (LC), one of the most prevalent malignancy, accounts for the highest number of cancer-related deaths, with nearly 1.8 million deaths reported globally in 2022 (1). Furthermore, based on the global analysis in 2021, LC ranked as the seventh leading cause of death and the seventeenth leading cause of disability-adjusted life-years (DALYs) (2). Consequently, strategies aimed at prevention and health promotion are essential for mitigating the global burden of LC.

LC comprises two major histological subtypes, namely non-small cell lung cancer (NSCLC) and small cell lung cancer (SCLC). These subtypes possess both similar and distinct etiologies and mutable risk factors, including smoking, metabolism, and diet (3–5). Concerning the risk factors related to LC, smoking is the most prominent. Owing to effective tobacco control strategies in recent decades, the number of LC cases associated with smoking has decreased significantly. Whereas the number of LC cases among never-smokers has still increased (6–8). A growing body of evidence underscores the considerable impact that dietary and metabolic factors have on the burden of LC (9, 10).

Unhealthy dietary patterns and related metabolic diseases, such as diabetes mellitus (DM), represent a critical global public health challenge. With changes in eating habits, the incidence of diabetes is also on the rise. Notably, DM is rising globally as a consequence of shifting dietary habits (11, 12). According to global statistics of disease-related risk factors from 1990 to 2021, high fasting plasma glucose (HFPG) as the metabolic risk factor has ascended from the 9th position to the 5th, while diet low in fruits (DLF) as the dietary risk factor had moved up from the 18th position to the 12th (2).

The epidemiological characteristics of the LC burden attributable to dietary and metabolic factors at global, regional, and national levels remain inadequately elucidated. Initiated in 1990, the Global Burden of Disease (GBD) study aims to assess health outcomes in a timely, effective, and relevant manner across 204 countries and regions (2, 13, 14). In this investigation, we utilized the latest GBD 2021 to quantify the LC burden attributable to diet and metabolism. Our analysis considered variables such as country, gender, and age to provide critical insights for formulating strategies aimed at preventing and controlling LC.

## 2 Materials and methods

### 2.1 Study data

For our study, we acquired annual data spanning from 1990 to 2021. The data encompassed information on deaths, DALYs, age-standardized mortality rates (ASMRs) and age-standardized DALYs rates (ASDRs) of LC attributable to metabolic and dietary risk factors based on socio-demographic index (SDI), location, age, and sex from GBD 2021.^1^ All metrics were reported with 95% uncertainty intervals (UIs). Additionally, population estimates for the years 2017 to 2,100 were also obtained from GBD 2021 (13, 15).

The Institutional Review Board at the University of Washington has thoroughly reviewed and granted an exemption for informed consent in relation to the GBD 2021 study (2). This research is reported in accordance with the STROCSS standards (16).

### 2.2 Definitions

In this study, LC is coded as C33–C34 in accordance with the 10th revision of the International Statistical Classification of Diseases and Related Health Problems (ICD-10).

By conducting a comprehensive literature review on risk factors associated with the global burden of LC, we specifically focused on metabolic factors, such as HFPG, and dietary factors, such as DLF (15). These established risk factors for LC were selected based on their availability in the GBD 2021. HFPG is defined as a fasting plasma glucose level exceeding 4.9–5.3 mmol/L. DLF is defined as an average daily consumption of less than 340–350 grams of fruit, encompassing fresh, frozen, cooked, canned, or dried fruit, with the exclusion of juice drinks and salt-preserved or pickled fruits.

The SDI serves as an indicator for assessing the level of development, encompassing average income per person, average education level, fertility rate, and other relevant data. Each country (or region) is assigned an SDI value ranging from 0 to 1, with higher values indicating a higher level of development. There are 204 countries or regions worldwide, each corresponding to a specific SDI value. The SDI is commonly categorized into five levels: low (41 countries), low-middle (41 countries), middle (40 countries), high-middle (41 countries), and high (41 countries) (2).

### 2.3 Estimated annual percentage change

The estimated annual percentage change (EAPC) is used to depict the patterns in the global burden of LC attributable to risk factors from 1990 to 2021. A linear relationship between the napierian logarithmic of the rate and time is established through the equation y = *α* + *β*x + *ε*, where x represents the year and y represents ln(rate). The EAPC is calculated as 100 × (e^*β*-1), with a 95% confidence interval (CI) provided (16, 17).

When EAPC and the lower limit of 95% CI are both greater than 0, it indicates an upward trend. Conversely, if the EAPC value and the upper limit of its 95% CI are both less than 0, it indicates a downward trend. Otherwise, the trend is considered stable.

### 2.4 Prediction model

The autoregressive integrated moving average (ARIMA) model is a statistical analysis model that combines Autoregressive and Moving Average models, along with an integrated differencing component (18, 19). It utilizes past values to predict future values and is classified as a type of time series method. Utilizing Global Burden of Disease (GBD) data spanning from 1990 to 2021, the ARIMA model was employed to project disease burdens up to 2036.

The parameters of the ARIMA model are denoted as ARIMA (p, d, q), where p represents the number of autoregressive terms, d indicates the degree of differencing required to achieve stationarity, and q signifies the number of lagged forecast errors in the prediction equation. The selection of optimal p and q values is guided by evaluating the akaike information criterion (AIC), corrected akaike information criterion (AICc), and bayesian information criterion (BIC). Additionally, assessing the training set’s performance using the root mean square error (RMSE) provides insight into the model’s predictive accuracy, while an autocorrelation function at lag 1 (ACF1) close to zero suggests that the model is appropriately specified.

### 2.5 Statistical analysis

The utilization of ASMR and ASDR allows for the comparison of mortality and DALY rates across countries with varying age compositions and demographic characteristics. In addition, all age-standardized rates (ASRs) are calculated per 100,000 population (20, 21). The Pearson correlation coefficient is used to investigate the relationship between SDI and ASRs of LC attributable to metabolic and dietary risk factors.

The data for this study were processed, analyzed, and visualized using R 4.3.2 (R Core Team, Vienna, Austria).

## 3 Results

### 3.1 Trends of LC burden attributable to HFPG

The global death number of LC attributed to HFPG was almost triple from three decades ago (from 0.019 million in 1990 to 0.051 million in 2021), accompanied by an observed increase in the ASMR (EAPC: 0.67, 95%CI: 0.52 to 0.83) from 0.49 to 0.59 (per 100,000 population) (Table 1). In other words, approximately 0.59% of LC-related deaths were attributed to HFPG in 2021. In addition, the global DALYs number of LC attributed to HFPG was more than doubled from 1990 to 2021, accompanied by a moderate increasing trend in the ASDR (EAPC: 0.39, 95%CI: 0.26 to 0.52) (Table 2). Among above metrics, the male-to-female ratios were both approximately 2.5 in 2021.

**Table 1:** Deaths and ASMR of lung cancer attributable to high fasting plasma glucose in 1990 and 2021, and EAPC of ASMR from 1990 to 2021.

Characteristics	1990		2021		1990–2021
	Number of deaths (95%UI)	ASMR per 100,000 (95%UI)	Number of deaths (95%UI)	ASMR per 100,000 (95%UI)	EAPC of ASMR (95%CI)
Global	18,976.61 (−3,911.01 to 41,995.18)	0.49 (−0.10 to 1.10)	50,546.90 (−10,046.30 to 115,605.54)	0.59 (−0.12 to 1.35)	0.67 (0.52 to 0.83)
Sex					
Male	14,163.92 (−2,873.96 to 31,397.40)	0.84 (−0.17 to 1.86)	34,000.57 (−6,748.94 to 78,316.05)	0.88 (−0.17 to 2.02)	0.31 (0.15 to 0.46)
Female	4,812.70 (−1,037.05 to 10,796.61)	0.23 (−0.05 to 0.51)	16,546.33 (−3,297.37 to 37,447.50)	0.35 (−0.07 to 0.80)	1.41 (1.24 to 1.58)
SDI region					
High SDI	8,563.66 (−1,757.88 to 18,794.57)	0.76 (−0.16 to 1.66)	18,072.95 (−3,630.90 to 40,820.67)	0.81 (−0.16 to 1.82)	0.23 (0.03 to 0.43)
High-middle SDI	5,660.15 (−1,156.31 to 12,661.58)	0.57 (−0.12 to 1.27)	15,090.48 (−3,035.45 to 34,298.62)	0.75 (−0.15 to 1.70)	1.09 (0.91 to 1.26)
Middle SDI	3,708.62 (−774.62 to 8,252.99)	0.39 (−0.08 to 0.87)	13,355.63 (−2,614.92 to 30,953.10)	0.51 (−0.10 to 1.19)	1.04 (0.93 to 1.16)
Low-middle SDI	824.30 (−171.41 to 1,796.51)	0.15 (−0.03 to 0.32)	3,372.49 (−646.99 to 7,711.91)	0.25 (−0.05 to 0.56)	1.77 (1.74 to 1.80)
Low SDI	192.62 (−38.88 to 431.82)	0.09 (−0.02 to 0.21)	594.88 (−111.83 to 1,320.48)	0.13 (−0.02 to 0.29)	1.06 (0.97 to 1.16)

ASMR, age-standardized mortality rate; CI, confidential interval; EAPC, estimated annual percentage change; SDI, sociodemographic index; UI, uncertainty interval.

**Table 2:** DALYs and ASDR of lung cancer attributable to high fasting plasma glucose in 1990 and 2021, and EAPC of ASDR from 1990 to 2021.

Characteristics	1990		2021		1990–2021
	Number of DALYs (95%UI)	ASDR per 100,000 (95%UI)	Number of DALYs (95%UI)	ASDR per 100,000 (95%UI)	EAPC of ASDR (95%CI)
Global	455,679.34 (−93,890.52 to 1,006,142.59)	11.33 (−2.34 to 25.05)	1,085,022.95 (−216,348.64 to 2,493,471.39)	12.43 (−2.48 to 28.54)	0.39 (0.26 to 0.52)
Sex					
Male	345,026.9 (−69,879.93 to 758,151.90)	18.64 (−3.78 to 41.19)	741,592.98 (−147,696.7 to 1,701,245.48)	18.21 (−3.63 to 41.86)	0.05 (−0.08 to 0.18)
Female	110,652.44 (−24,010.58 to 250,054.73)	5.16 (−1.12 to 11.66)	343,429.97 (−68,764.7 to 777,314.58)	7.41 (−1.48 to 16.77)	1.17 (1.02 to 1.31)
SDI region					
High SDI	193,061.13 (−39,665.34 to 423,164.75)	17.48 (−3.59 to 38.38)	347,423.64 (−70,133.23 to 781,437.06)	16.61 (−3.36 to 37.24)	−0.14 (−0.33 to 0.04)
High-middle SDI	141,894.55 (−28,852.97 to 319,090.41)	13.79 (−2.81 to 30.98)	334,729.42 (−67,415.17 to 759,647.56)	16.55 (−3.34 to 37.56)	0.76 (0.61 to 0.92)
Middle SDI	93,938.58 (−19,671.34 to 208,713.28)	8.90 (−1.86 to 19.77)	303,705.26 (−59,830.65 to 702,205.72)	11.07 (−2.18 to 25.62)	0.83 (0.73 to 0.93)
Low-middle SDI	21,140.70 (−4,426.12 to 45,569.90)	3.40 (−0.71 to 7.38)	82,911.07 (−16,043.68 to 189,192.47)	5.65 (−1.09 to 12.91)	1.72 (1.70 to 1.75)
Low SDI	4,974.69 (−1,008.11 to 11,172.22)	2.16 (−0.44 to 4.83)	14,919.82 (−2,824.95 to 33,222.74)	2.93 (−0.55 to 6.51)	0.93 (0.83 to 1.03)

ASDR, age-standardized DALYs rate; CI, confidential interval; DALY, disability-adjusted life-year; EAPC, estimated annual percentage change; SDI, sociodemographic index; UI, uncertainty interval.

ASMR (0.81 per 100,000 population) and ASDR (16.61 per 100,000 population) were also highest in the high SDI regions, whereas EAPC in the high SDI regions showed a slight increase in ASMR and ASDR over the 30 years. Nevertheless, ASMR (EAPC: 1.77, 95%CI: 1.74 to 1.80) and ASDR (EAPC: 1.72, 95%CI: 1.70 to 1.75) experienced the most increase in the low-middle SDI regions (Tables 1, 2).

**Figure 1:**
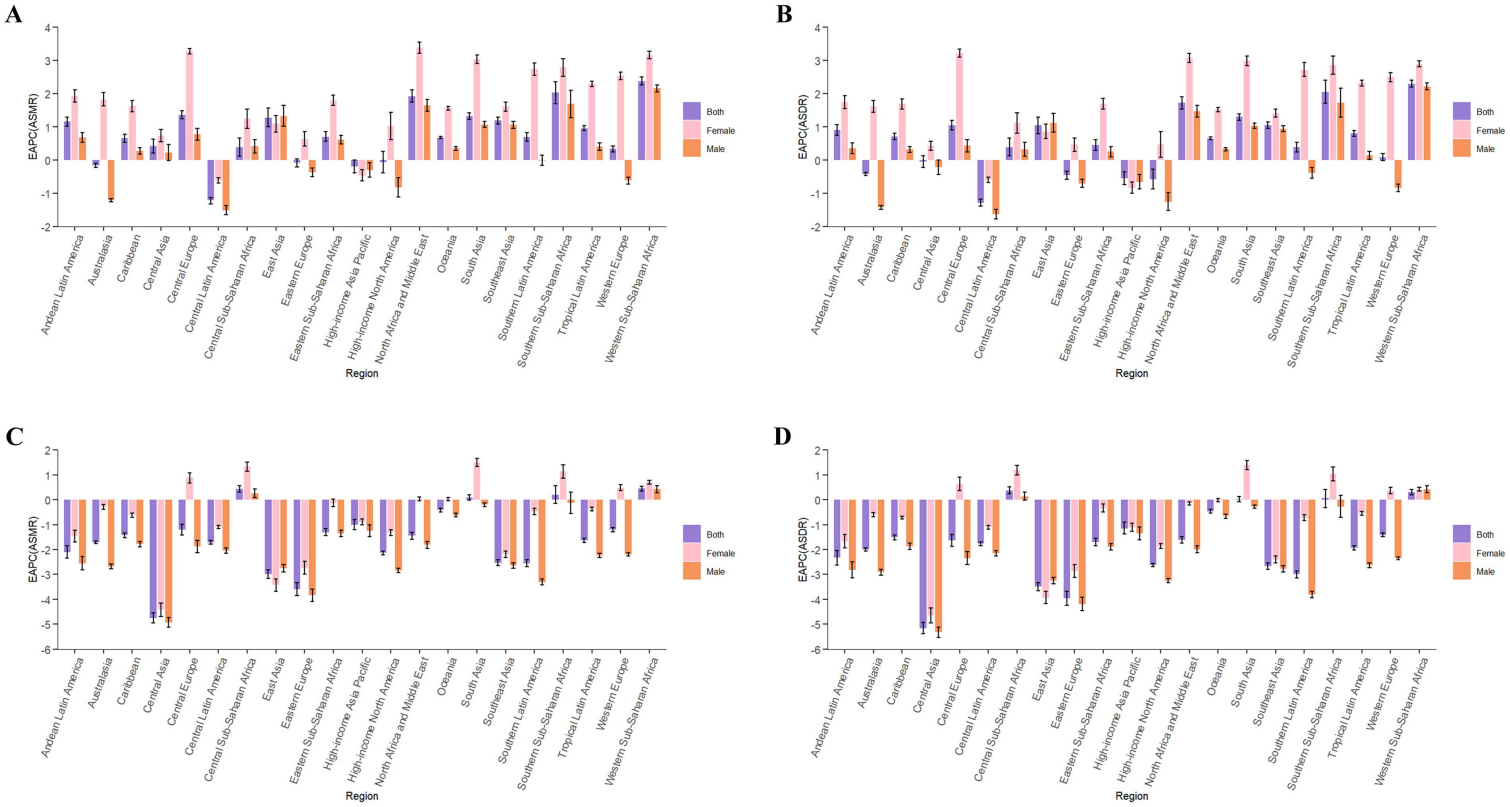
EAPC of ASMR **(A)** and ASDR **(B)** of lung cancer attributable to high fasting plasma glucose from 1990 to 2021, by sex, in 21 regions. EAPC of ASMR **(C)** and ASDR **(D)** of lung cancer attributable to diet low in fruits from 1990 to 2021, by sex, in 21 regions. ASMR, age-standardized mortality rate; ASDR, age-standardized DALYs rate; DALYs, disability-adjusted life-years; EAPC, estimated annual percentage change.

For the GBD regions, LC-related ASMR and ASDR attributable to HFPG increased in 15 GBD regions (Supplementary Figure S2). The East Asia region had the highest number of death cases and DALYs (0.017 million and 0.397 million) in 2021, as a percentage over 35 worldwide (Supplementary Tables S1, S2). Western Sub-Saharan Africa had the greatest increase in ASMR (EAPC:2.38, 95%CI: 2.27 to 2.50) and ASDR (EAPC:2.30, 95%CI: 2.20 to 2.41) from 1990 to 2021. Notably, Western Sub-Saharan Africa, Southern Sub-Saharan Africa, and North Africa and Middle East ranked in the top three regions, as all EAPCs greater than 1.5 (Figures 1A,B).

**Figure 2:**
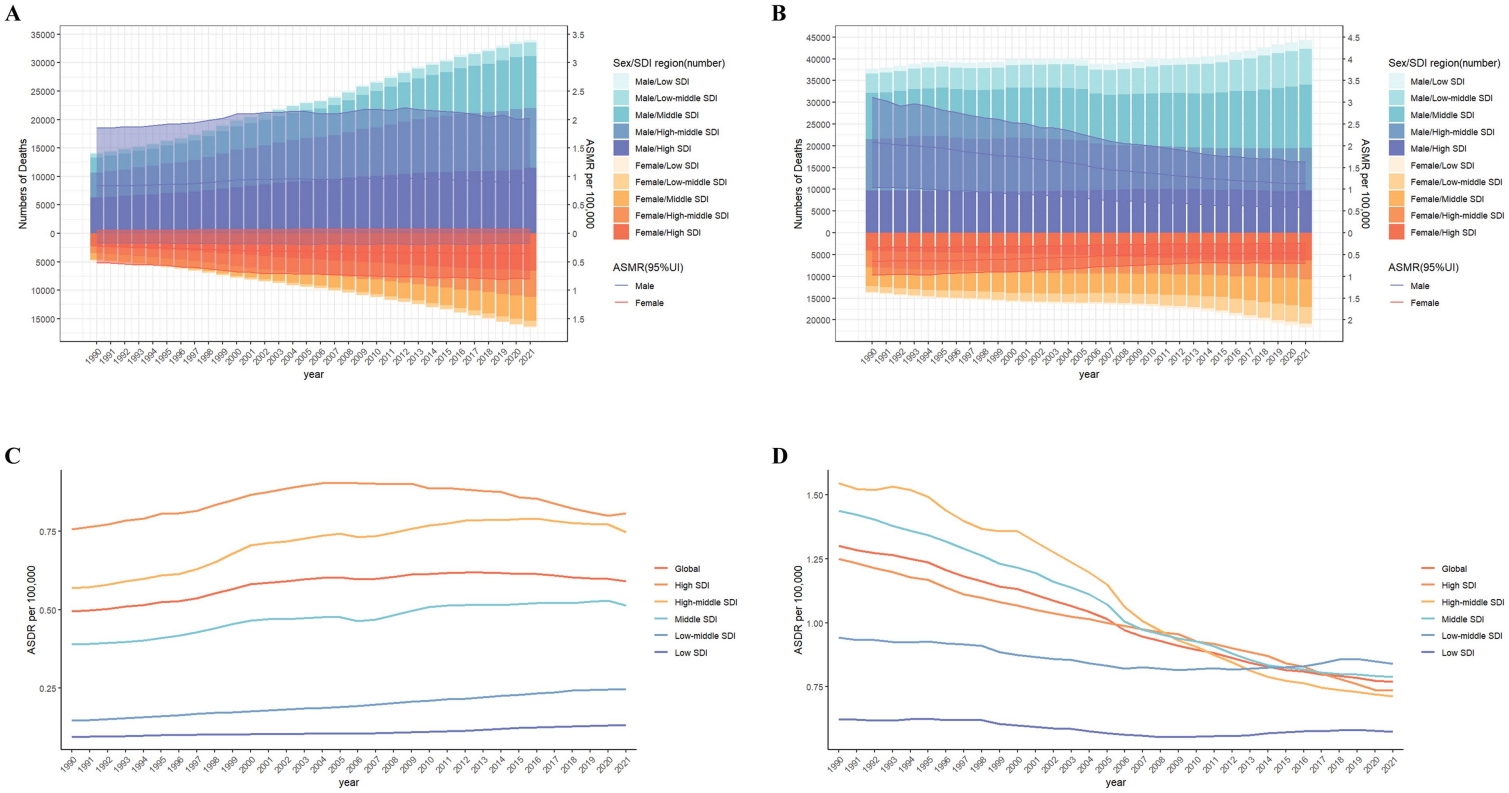
The burden of lung cancer deaths attributable to high fasting plasma glucose **(A)** and diet low in fruits **(B)** from 1990 to 2021 by sex and SDI region. The bars display the number of lung cancer deaths. The lines with 95% UI represent ASMR. SDI regional ASMR of lung cancer attributable to high fasting plasma glucose **(C)** and diet low in fruits **(D)** from 1990 to 2021. ASMR, age-standardized mortality rate; SDI, sociodemographic index; UI, uncertainty interval.

SDI regionally, in 2021, the numbers of LC-related death cases (0.018 million) and DALYs (0.35 million) attributable to HFPG in the high SDI regions were the highest (Figure 2). The ASMR.

**Figure 3:**
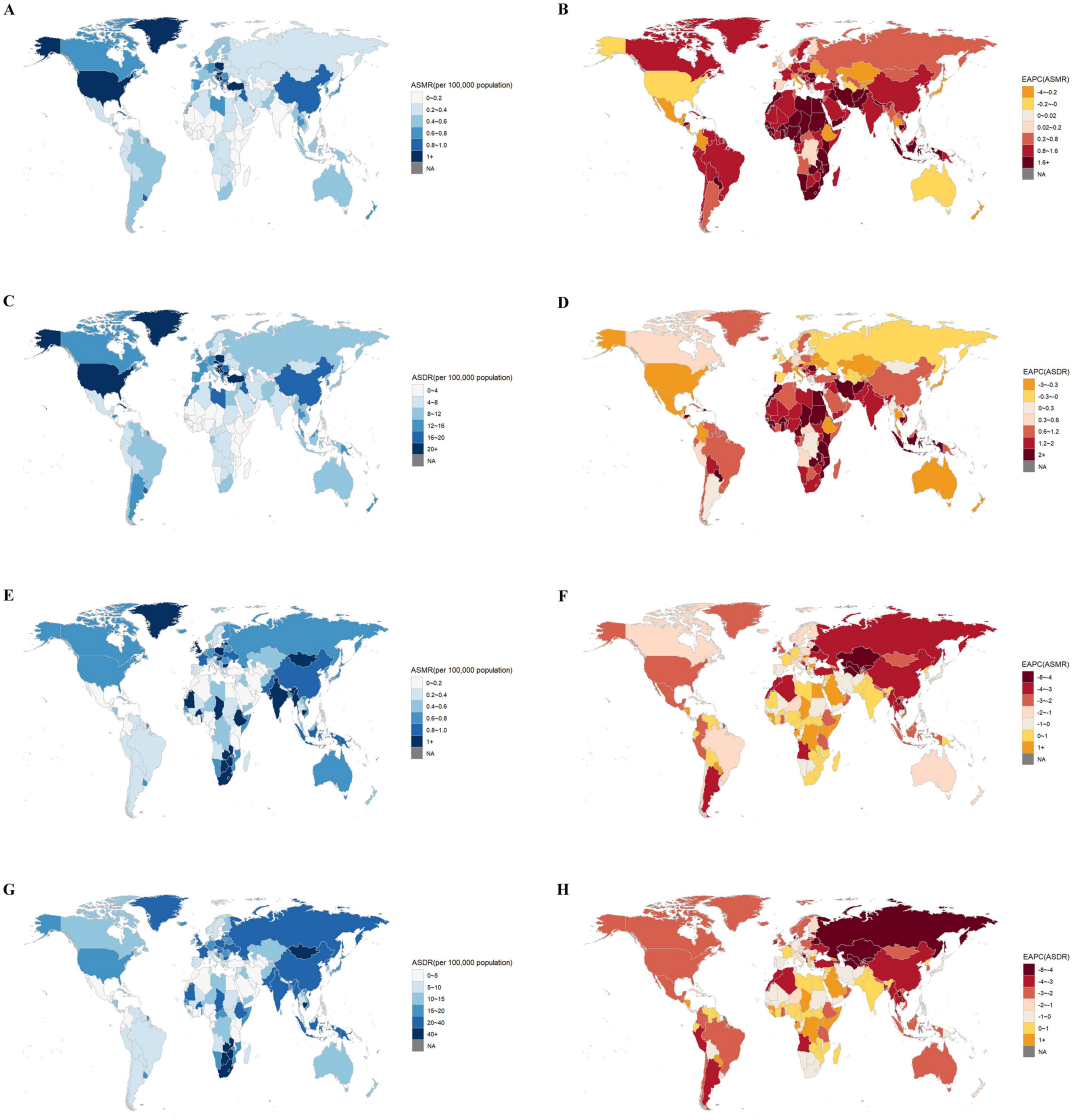
The global distribution of ASMR **(A)** and ASDR **(C)** of lung cancer attributable to high fasting plasma glucose in 2021, and EAPC in ASMR **(B)** and ASDR **(D)** of lung cancer attributable to high fasting plasma glucose from 1990 to 2021. The global distribution of ASMR **(E)** and ASDR **(G)** of lung cancer attributable to diet low in fruits in 2021, and EAPC in ASMR **(F)** and ASDR **(H)** of lung cancer attributable to diet low in fruits from 1990 to 2021. ASMR, age-standardized mortality rate; ASDR, age-standardized DALYs rate; DALYs, disability-adjusted life-years; EAPC, estimated annual percentage change.

At the 204 countries, China had the highest number of death cases and DALYs (0.017 million and 0.39 million). In ASMR and ASDR, Palau, Marshall Islands, Northern Mariana Islands, Montenegro and Nauru ranked in the top five countries in 2021 (Figures 3A,C). A remarkable thing was that Egypt had experienced the most rapid increase in ASMR (EAPC: 7.84, 95%CI: 7.11 to 8.58) and ASDR (EAPC: 7.35, 95%CI: 6.70 to 8.00) (Figures 3B,D).

### 3.2 Trends of LC burden attributable to DLF

Globally, the numbers of death cases (from 0.051million in 1990 to 0.066 million in 2021) and DALYs (from 1.44 million in 1990 to 1.61 million 2021) for LC attributable to DLF exhibited a slight increase. Nevertheless, ASMR (EAPC: -1.89, 95%CI: −1.96 to −1.82) and ASDR (EAPC: -2.23, 95%CI: −2.31 to −2.14) were both decreasing from 1990 to 2021 (Tables 3, 4). The male-to-female ratio in these indicators was both approximately 2 in 2021. Additionally, the EAPCs of ASMR and ASDR were slightly greater for male compare to female.

**Table 3:** Deaths and ASMR of lung cancer attributable to diet low in fruits in 1990 and 2021, and EAPC of ASMR from 1990 to 2021.

Characteristics	1990		2021		1990–2021
	Number of deaths (95%UI)	ASMR per 100,000 (95%UI)	Number of deaths (95%UI)	ASMR per 100,000 (95%UI)	EAPC of ASMR (95%CI)
Global	51,621.37 (25,769.75 to 75,860.15)	1.3 (0.65 to 1.91)	66,045.46 (34,005.89 to 97,033.46)	0.77 (0.40 to 1.13)	−1.89 (−1.96 to −1.82)
Sex					
Male	37,726.67 (18,866.62 to 56,539.44)	2.08 (1.05 to 3.11)	44,328.29 (22,872.11 to 64,126.67)	1.12 (0.58 to 1.63)	−2.15 (−2.21 to −2.09)
Female	13,894.70 (7,255.52 to 20,690.59)	0.65 (0.34 to 0.97)	21,717.17 (11,100.42 to 32,326.79)	0.47 (0.24 to 0.70)	−1.32 (−1.41 to −1.22)
SDI region					
High SDI	13,872.00 (7,056.80 to 20,227.43)	1.25 (0.64 to 1.82)	16,064.47 (8,039.53 to 24,167.47)	0.73 (0.37 to 1.10)	−1.64 (−1.71 to −1.57)
High-middle SDI	15,652.67 (7,764.87 to 23,387.83)	1.55 (0.77 to 2.31)	14,174.69 (7,071.55 to 21,347.38)	0.71 (0.36 to 1.07)	−2.93 (−3.10 to −2.75)
Middle SDI	14,842.70 (7,433.52 to 22,198.23)	1.44 (0.72 to 2.15)	20,847.49 (10,804.29 to 30,841.29)	0.79 (0.41 to 1.17)	−2.21 (−2.31 to −2.10)
Low-middle SDI	5,794.91 (3,055.61 to 8,625.02)	0.94 (0.50 to 1.41)	12,105.29 (6,258.33 to 17,014.52)	0.84 (0.44 to 1.18)	−0.42 (−0.53 to −0.31)
Low SDI	1,409.76 (744.02 to 2,181.23)	0.62 (0.33 to 0.96)	2,800.08 (1,383.32 to 3,997.61)	0.57 (0.28 to 0.82)	−0.35 (−0.45 to −0.25)

ASMR, age-standardized mortality rate; CI, confidential interval; EAPC, estimated annual percentage change; SDI, sociodemographic index; UI, uncertainty interval.

**Table 4:** DALYs and ASDR of lung cancer attributable to diet low in fruits in 1990 and 2021, and EAPC of ASDR from 1990 to 202.

Characteristics	1990		2021		1990–2021
	Number of DALYs (95%UI)	ASDR per 100,000 (95%UI)	Number of DALYs (95%UI)	ASDR per 100,000 (95%UI)	EAPC of ASDR (95%CI)
Global	1,435,375.14 (721,498.80 to 2,119,984.28)	34.39 (17.24 to 50.70)	1,611,267.04 (828,053.56 to 2,347,369.49)	18.46 (9.49 to 26.90)	−2.23 (−2.31 to −2.14)
Sex					
Male	1,059,478.5 (534,061.42 to 1,599,531.77)	53.64 (27.01 to 80.74)	1,097,919.60 (566,432.74 to 1,597,498.78)	26.48 (13.66 to 38.50)	−2.47 (−2.54 to −2.40)
Female	375,896.64 (196,209.09 to 560,465.64)	17.22 (8.98 to 25.64)	513,347.43 (261,758.99 to 754,495.90)	11.26 (5.74 to 16.55)	−1.64 (−1.75 to −1.53)
SDI region					
High SDI	336,402.13 (170,223.99 to 487,385.34)	31.18 (15.77 to 45.16)	322,708.77 (163,061.57 to 479,678.81)	16.18 (8.21 to 24.09)	−2.03 (−2.11 to −1.95)
High-middle SDI	451,238.24 (225,372.62 to 672,449.64)	43.32 (21.63 to 64.65)	338,956.38 (167,188.23 to 513,037.21)	17.17 (8.48 to 25.96)	−3.46 (−3.64 to −3.27)
Middle SDI	435,241.92 (214,869.03 to 649,544.74)	37.98 (18.93 to 56.70)	529,165.89 (271,427.98 to 776,882.84)	18.97 (9.76 to 27.83)	−2.53 (−2.65 to −2.41)
Low-middle SDI	169,941.35 (89,659.36 to 252,673.31)	25.21 (13.29 to 37.46)	339,645.21 (176,183.28 to 478,077.58)	21.96 (11.38 to 30.93)	−0.47 (−0.59 to −0.36)
Low SDI	41,193.83 (21,754.85 to 63,799.66)	16.45 (8.68 to 25.52)	79,513.10 (39,178.95 to 113,065.90)	14.28 (7.06 to 20.35)	−0.57 (−0.68 to −0.47)

ASDR, age-standardized DALYs rate; CI, confidential interval; DALY, disability-adjusted life-year; EAPC, estimated annual percentage change; SDI, sociodemographic index; UI, uncertainty interval.

At the SDI region level, the highest numbers of LC-related deaths and DALYs attributable to DLF was experienced in the middle SDI (0.021 million and 0.53 million), as a percentage over 30 worldwide. Notably, high-middle SDI and middle SDI evidenced a more rapid descent in ASMR and ASDR, with all EAPCs greater than two from 1990 to 2021 (Tables 3, 4).

At the 21 GBD regions, LC-related ASMR and ASDR attributable to DLF decreased in 17 GBD regions. East Asia had the highest number of deaths (0.020 million), whereas South Asia had the highest number of DALYs (0.47 million) in 2021 (Supplementary Figure S2). From 1990 to 2021, Central Asia had the greatest decrease in ASMR (EAPC: -4.75, 95%CI: −4.96 to −4.54) and ASDR (EAPC: -5.16, 95%CI: −5.38 to −4.93) (Figures 1C,D). Central Asia, Eastern Europe and East Asia ranked in the top three regions, with all upper limit of EAPCs greater than 3 (Supplementary Tables S3, S4).

For the countries, China had the highest number of deaths (0.019 million) and DALYs (0.44 million). Mongolia, Lesotho, Zimbabwe, Cambodia and Zambia ranked in the top five countries for ASMR and ASDR in 2021 (Figures 3E,G). A remarkable thing was that Kazakhstan had experienced the most rapid decrease in both ASMR (EAPC: -7.42, 95%CI: −7.79 to −7.04) and ASDR (EAPC: −7.85 95%CI: −8.25 to −7.44) (Figures 3F,H).

### 3.3 Age-specific global LC burden attributable to HFPG and DLF

**Figure 4:**
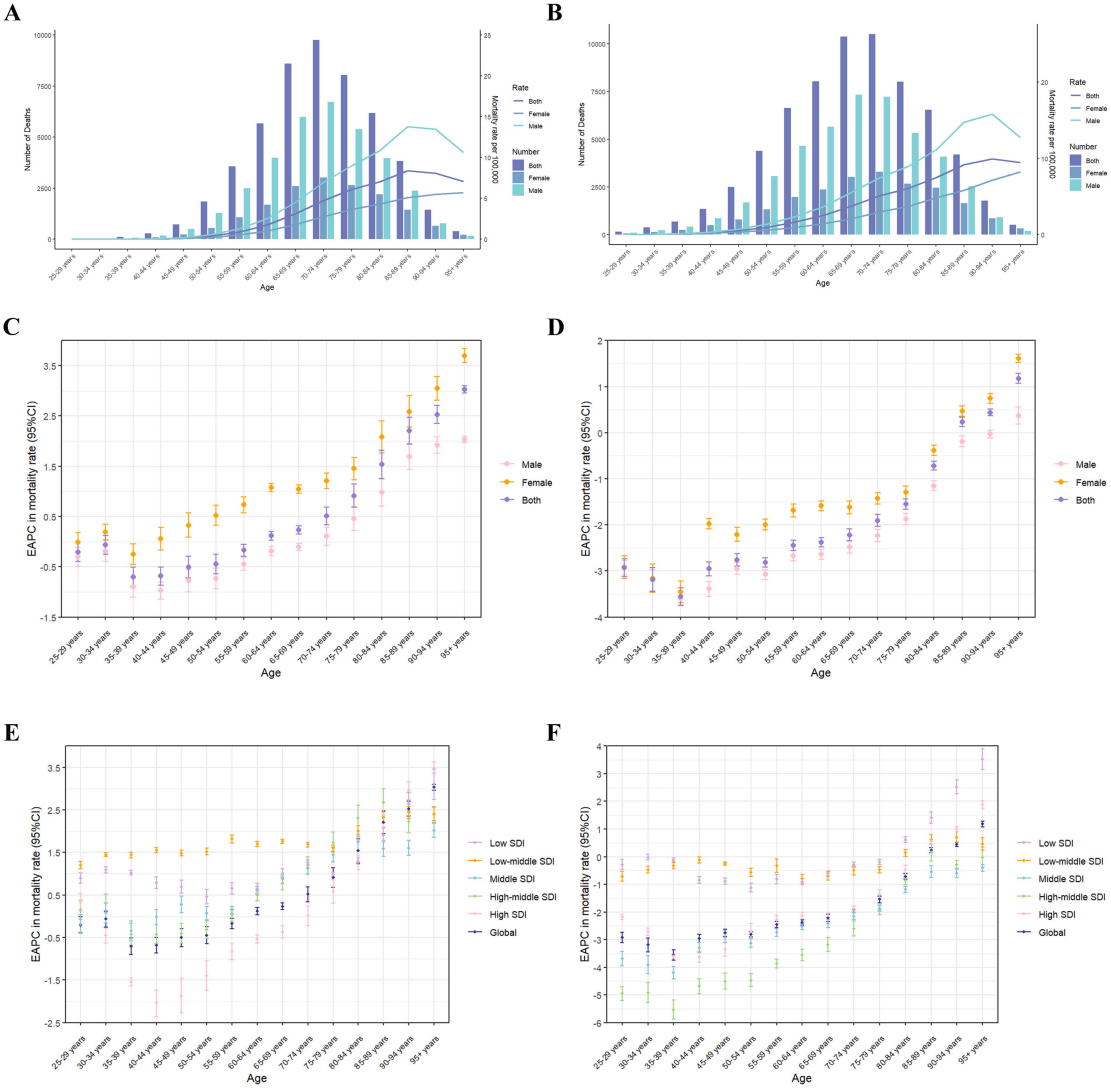
The age distribution of lung cancer deaths attributable to high fasting plasma glucose **(A)** and diet low in fruits **(B)** in different age groups, by sex, in 2021. The bars display the number of lung cancer deaths. The lines represent ASMR. EAPC of global mortality rates of lung cancer attributable to high fasting plasma glucose in different age groups from 1990 to 2021, by sex **(C)** and SDI region **(E)**. EAPC of global mortality rates of lung cancer attributable to diet low in fruits in different age groups from 1990 to 2021, by sex **(D)** and SDI region **(F)**. ASMR, age-standardized mortality rate; EAPC, estimated annual percentage change; CI, certainty interval; SDI, sociodemographic index.

The age distribution of death cases related to HFPG globally in 2021, as shown in Figure 4A, exhibits an inverted V-shaped pattern, with the greatest amount of deaths attributable to HFPG occurring the age group ranging from 70 to 74. The mortality rates exhibit synchronous patterns of increase and subsequent decrease across various age groups for both genders. This discovery highlights that age is a crucial factor in the influence of the global LC burden attributable to HFPG. The mortality rate of male peaks in the 85 to 89 years old and then gradually declines, whereas the mortality rate of female shows an upward trend. Notably, the mortality among males is considerably higher than females, with the gender difference in mortality rates being most evident in the age group of 85–89. The age distribution of HFPG-related DALYs and DALY rates, as illustrated, demonstrates a similar trend (Supplementary Figure S3A). Notably, there is a prominent peak in DALYs within the 65 to 69 age group, while the highest rate of DALYs occurs among individuals aged 75 to 79 (Figure 4B).

Globally, from 1990 to 2021, EAPCs of the mortality rate related to HFPG for the age groups under 60 years old are less than 0, whereas EAPCs are greater than 0 for the age group above 60 years old. For the SDI regions, it is notable that in the lower SDI regions, the mortality rates of each age group have been increasing, as all EAPCs being greater than 0. Furthermore, in the high SDI regions, the EAPC shows a V-shaped distribution, while the age group of 40–44 witnesses the fastest decline of mortality rate (Figures 4C,E). The EAPCs of the specific age DALYs ratios have demonstrated similar patterns of change (Supplementary Figures S3C,E).

At the global level, the age distribution pattern of the global burden attributable to DLF is similar in 2021 (Figure 4A; Supplementary Figure S3B). Whereas, the gender disparity in mortality is most prominent in the age group of 90 to 94, and the peak of the DALY rate is in the 70 to 74 age group (Supplementary Figures S3D,F). From 1990 to 2021, the EAPCs for all age groups under 85 are less than 0. The EAPCs for middle SDI regions are less than 0. The EAPCs for mortality in middle SDI, high-middle SDI, and high SDI regions both show a V-shaped distribution. Notably, the decline rate is the fastest in the 35 to 39 age group (EAPCs are greater than 3), and the EAPCs for all age groups under 85 are less than 0 (Figures 4D,F). The EAPCs for the specific age of DALYs ratios demonstrate similar patterns.

### 3.4 Associations with the SDI

**Figure 5:**
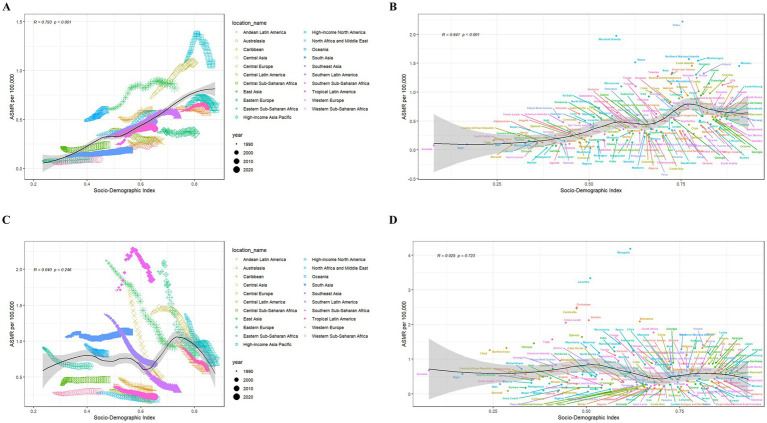
Correlations between ASMR of lung cancer attributable to high fasting plasma glucose and SDI at the regional **(A)** and national **(B)** level. Correlations between ASMR of lung cancer attributable to diet low in fruits and SDI at the regional **(C)** and national **(D)** level. The black line represents the expected ASMR on the basis of SDIs in all locations. ASMR, age-standardized mortality rate; SDI, sociodemographic index.

The correlations between ASMR of LC attributable to HFPG and SDI at regional and national level are observed to be positive (R = 0.793, *p* < 0.001 for region, R = 0.641, *p* < 0.001 for country) (Figure 5). Similarly, ASDR is positively correlated with SDI (R = 0.795, *p* < 0.001 for region, R = 0.618, *p* < 0.001 for country) (Supplementary Figure S4). Additionally, High-income North America, Central Europe, East Asia, and Oceania have observed higher ASMR and ASDR than expected in accordance with their SDI. At the national level, some countries, such as Palau, Marshall Islands and Nauru, have observed significantly higher ASMR and ASDR than expected in accordance with their SDI. In addition, our analysis shows no correlation between SDI and ASMR of LC attributable to DLF.

### 3.5 Global burden predictions for LC attributable to HFPG and DLF

**Figure 6:**
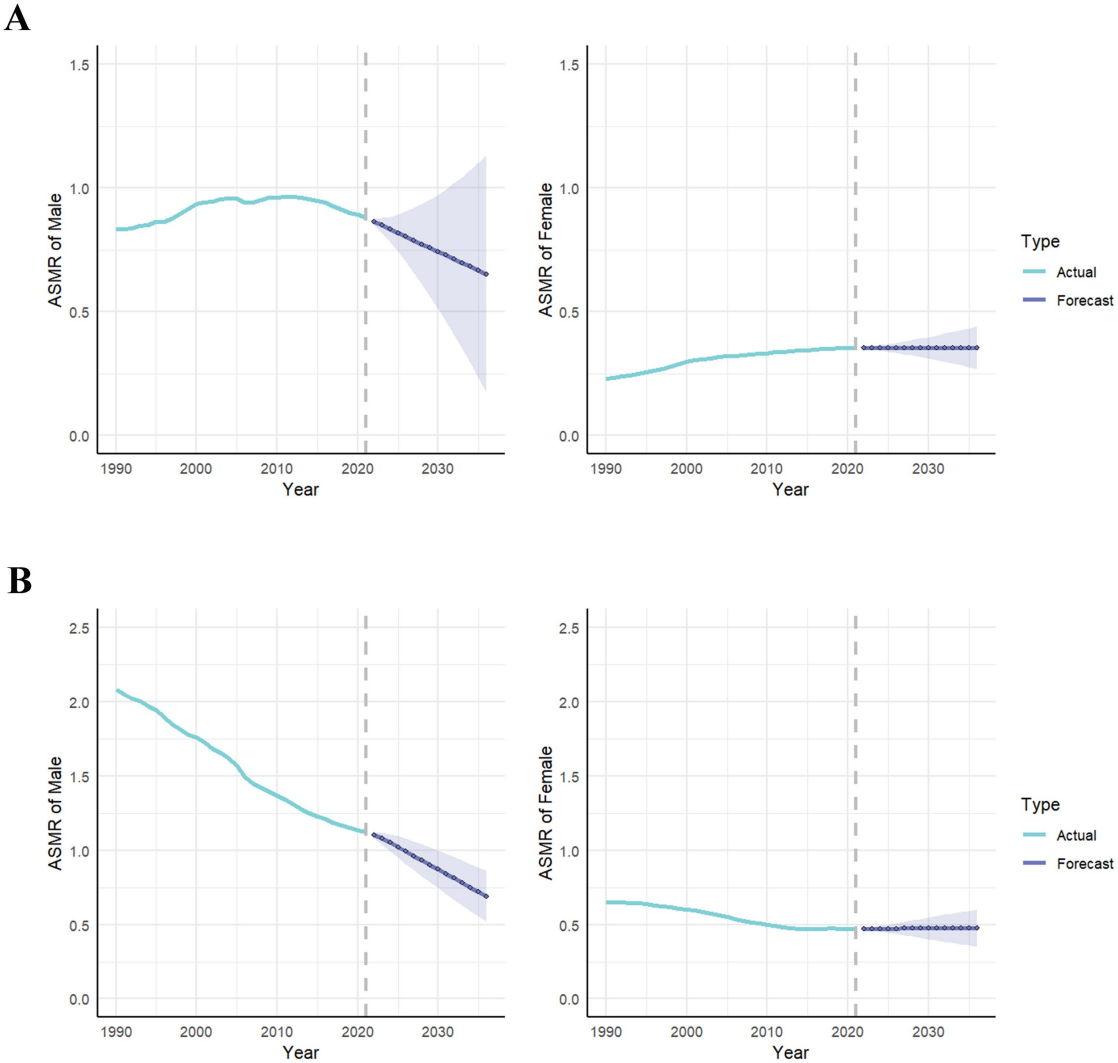
The actual and predicted values in ASMR of lung cancer attributable to high fasting plasma glucose **(A)** and diet low in fruits **(B)** with the ARIMA model by sex. ASMR, age-standardized mortality rate; ARIMA, autoregressive integrated moving average.

The ARIMA model was established to predict LC risk–attributable burden in next 15 years. Supplementary Table S5 shows the parameters of ARIMA model. Our research shows the prediction trends of LC attributable to HFPG in ASMR and ASDR from 1990 to 2036, by gender and the SDI region (Figure 6; Supplementary Figure S5). Globally, the predictive results indicate that ASMR and ASDR will undergo a gradual decline in the next 15 years. Notably, with exception of high-middle SDI regions, ASMR will continue to increase in all other SDI regions. Interestingly, ASMR of male will continue to decline, whereas ASMR of female will moderate increase. It suggests that both gender and SDI are important factors.

**Figure 7:**
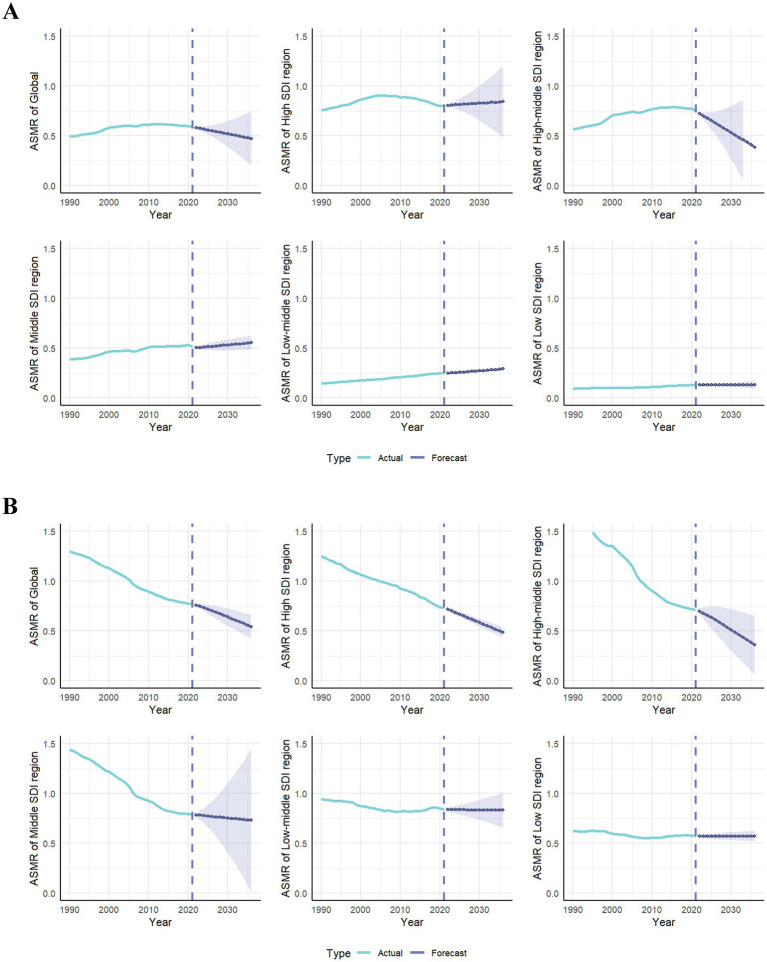
The actual and predicted values in ASMR of lung cancer attributable to high fasting plasma glucose **(A)** and diet low in fruits **(B)** with the ARIMA model by global and SDI regions. ASMR, age-standardized mortality rate; ARIMA, autoregressive integrated moving average; SDI, sociodemographic index.

According to Figure 7 and Supplementary Figure S6, it could be observed prediction of LC attributable to DLF from 1990 to 2036, by gender and the SDI region. Our predictive results indicate that all ASMR and ASDR attributable to DLF will continue to decline.

## 4 Discussion

### 4.1 Principal study findings

This study utilizes the latest GBD 2021 to thoroughly analyze burden of LC attributable to metabolic and dietary risk factors from 1990 to 2021. Furthermore, this study predicts trends for the next 15 years. From the GBD database, HFPG and DLF are identified as the sole metabolic and dietary risk factors for LC, respectively. The study findings indicate that a considerable increase in the LC burden attributable to HFPG, whereas ASMR and ASDR of LC attributable to DLF represent a general decline. There is an unequal distribution. In low-middle SDI regions, the ASMR and ASDR of LC related to DLF display the most rapid increase. Conversely, in the high-middle SDI regions, the LC burden related to DLF display the most rapid decline. Furthermore, the LC burden attributable to HFPG and DLF in mortality and DALYs is higher for males than females. The sex difference is more pronounced in the elderly. It is noteworthy for the significant disparities related to gender, age, and temporal–spatial patterns in our study.

Previous studies utilizing the GBD database have analyzed the global, regional, and national burden of LC (22–24) and its attributable risk factors (25). Notably, relevant GBD studies focused on specifically quantified the epidemiological characteristics of LC burden associated with particulate matter air pollution and smoking (18, 26, 27). As far as we know, our study represents the first attempt to specifically investigate the impact of dietary and metabolic factors on the burden of LC from 1990 to 2021 and future trends. Furthermore, our study not only supplements the previous research but also offers comprehensive insights for designing and promoting targeted prevention strategies against LC based on diet and metabolism.

### 4.2 Spatiotemporal pattern of LC burden attributable to metabolic and dietary risk factors

From 1990 to 2021, the burden of LC attributable to HFPG has escalated across most countries and regions, while the trends aligning with the incidence rate of DM. Over the past 3 decades, the global age-standardized prevalence rate of DM has surged by 90.5% (11). The SDI, as an indicator of socio-demographic development, exhibits a strong correlation with the burden of diseases. Furthermore, it’s a positive correlation between HFPG-related LC burden and SDI. High SDI region reports the highest numbers of deaths, along with DALYs, ASMR and ASDR, due to improvements in living standards (28), lifestyle changes, population aging (29, 30), and heightened awareness. Conversely, high SDI region exhibits the least pronounced upward trend in these metrics. These metrics in low-middle SDI region are less than one-third of those in high SDI region, whereas these demonstrate the most substantial growth rate in low-middle SDI region. With slower socioeconomic development, the management of DM is hindered by various health system factors (31), including a lack of knowledge and professional skills among healthcare professionals, limited accessibility and affordability of relevant supplies, inadequate availability of simple diagnostic and monitoring equipment, as well as the absence of locally tailored guidelines (32–34). It is concerning that the projected results indicate a continued gradual increase in the burden of HFPG-related LC across high SDI, middle SDI, low-middle SDI, and low SDI regions over the next 15 years.

Most countries and regions experience a significant decrease in ASMR and ASDR of the LC associated with DLF, while some regions maintain stability or show a slight increase. The most pronounced decline in the burden of LC is observed in high SDI region, whereas the reduction in lower SDI regions of LC associated with DLF remains negligible. At the regional and national level, due to a series of reforms, the healthcare system in Central Asia (35, 36) (including countries such as Kazakhstan) has achieved a degree of stability, leading to the most significant reduction in LC burden attributable to low fruit consumption. In contrast, the LC burden associated with DLF continues to rise across most of Africa. Notably, the Sub-Saharan African region exhibits the highest levels of ASMR and ASDR. In light of these disparities, policymakers in each country should formulate targeted strategies that address local risk factors to enhance balanced diets through increased fruit consumption, particularly in the majority of African countries (37, 38).

### 4.3 Patterns in gender and age groups of LC burden related to HFPG and DLF

The temporal trends of the LC burden related to HFPG and DLF follow similar patterns in gender and age groups. Over the past few decades, the baseline of mortality rate for lung cancer has been notably higher in males. These observations may be attributed to the unique biological characteristics and gender differences in LC. Female patients exhibit a higher frequency of EGFR mutations, particularly among non-smokers, which may render them more responsive to targeted therapies. In contrast, male patients are more frequently associated with KRAS mutations, leading to a poorer prognosis. Additionally, women tend to seek medical help more proactively, resulting in higher rates of early diagnosis and potentially better outcomes. The age-standardized prevalence ratio of DM between male and female is 1.14 (11), coupled with a lower daily fruit intake among males. Consequently, it elucidates the significantly higher mortality rates observed in males compared to females. For age demographics, the mortality rate is highest among 85 years and above. This can be interpreted that the elderly individuals have a declining pancreas function, resulting in weakened ability to regulate blood sugar, and have significantly lower fruit intake than young populations. In the higher SDI regions, the mortality rate among individuals under 50 years exhibits a significant decline trend. This phenomenon may be attributed to the relatively lower degree of exposure to risk factors during youth, which gradually escalates to its peak as individuals age. However, in low SDI and low-middle SDI regions, the reduction in the DLF-related mortality rates is minimal, while HFPG-related mortality rates are even observed to be on the rise. We hypothesize that in economically less developed countries or regions, young people’s unhealthy dietary patterns (39, 40) and low adherence to specific LC screening (41, 42) may be the main reasons. Consequently, it is imperative to prioritize the prevention and management of LC associated with DLF and HFPG among key demographic groups (43). Additionally, the point is that the increase in the mortality rate of female is greater than males in all age groups. The predicted results suggest that the ASMR and ASDR in females will still be slowly increasing in next 15 years. Over the past three decades, the disparity in LC burden between males and females has gradually narrowed, and it is predicted that this trend will continue in the future.

### 4.4 Public health implications

Fortunately, the dietary and metabolic risk factors related to LC are changeable, offering a chance to prevent this fatal cancer. By adjusting diet (44, 45), doing daily exercise appropriately, and using medication properly to prevent and improve an unhealthy condition of HFPG and DLF, it can be an auxiliary strategy to reduce the burden of LC. Our study emphasizes the importance of dealing with HFPG and DLF, which prompts policymakers to allocate resources more effectively, and national governments can develop strategies to face the challenges of the heavy LC burden based on dietary and metabolic risks at the national level.

### 4.5 Strengths and limitations

Our study has several limitations. Firstly, there exists heterogeneity in the prevalence trend of the two distinct histological subtypes of LC (SCLC and NSCLC). However, due to the unavailability of data within the GBD 2021, we were unable to conduct subtype analysis for different tissue types (46) in our study. Secondly, The GBD model lacks a comprehensive evaluation of the quality of its data sources and is further complicated by variations in monitoring systems across different countries, thereby potentially compromising the accuracy of its findings. Thirdly, temporal and spatial variations in LC burden associated with other dietary and metabolic risk factors, such as the intake of red meat, were not analyzed due to the unavailability data within the GBD 2021. There is a possibility that the burden of LC examined in our GBD study may be overestimated (47, 48). Finally, it should be advised that the GBD study is not intended for testing causality between different factors, therefore, inferring causality from any of the results presented in this study should be avoided unless further validation is conducted. Further comprehensive research is required to enhance the evidence and comprehend the mediating effects of gender, age, and geographic location on dietary and metabolic risks associated with LC burden.

## 5 Conclusion

In conclusion, regarding metabolic risk, the global burden of LC attributable to HFPG has risen in parallel with the increasing incidence of DM, over the past three decades. The elderly population, males, and regions with higher SDI experience a disproportionately greater disease burden. Conversely, more adverse trends have been observed in regions with lower SDI and among females. Moreover, it is anticipated that this burden will persistently increase across most regions over the next 15 years. Concerning dietary risk, the burden of LC attributable to DLF has shown a declining trend from 1990 to 2021, with sub-Saharan Africa, representing low SDI, exhibiting the highest ASMR and ASDR. Notably, this burden has decreased at a faster rate in regions with higher SDI. Additionally, gender disparities in the LC burden related to dietary and metabolic risk become increasingly pronounced with advancing age, whereas these differences are gradually diminishing over time. Our study aims to provide robust evidence supporting local governments in developing effective regulations for controlling and preventing LC.

## Data Availability

The original contributions presented in the study are included in the article/Supplementary material, further inquiries can be directed to the corresponding author.

## Footnotes

^1^ http://ghdx.healthdata.org/gbd-results-tool

## Supplementary material

SUPPLEMENTARY FIGURE S1 The burden of lung cancer DALYs attributable to high fasting plasma glucose **(A)** and diet low in fruits **(B)** from 1990 to 2021 by sex and SDI region. The bars display the number of lung cancer deaths. The lines with 95% UI represent ASMR. SDI regional ASDR of lung cancer attributable to high fasting plasma glucose **(C)** and diet low in fruits **(D)** from 1990 to 2021. ASDR, age-standardized DALYs rate; DALYs, disability-adjusted life-years; SDI, sociodemographic index; UI, uncertainty interval.

SUPPLEMENTARY FIGURE S2 ASMR **(A)** and ASDR **(B)** of lung cancer attributable to high fasting plasma glucose in 2021, by sex in 21 regions. ASMR **(C)** and ASDR **(D)** of lung cancer attributable to high fasting plasma glucose in 1990 and 2021 in 21 regions. ASMR **(E)** and ASDR **(F)** of lung cancer attributable to diet low in fruits in 2021, by sex in 21 regions. ASMR **(G)** and ASDR **(H)** of lung cancer attributable to diet low in fruits in 1990 and 2021 in 21 regions. ASMR, age-standardized mortality rate; ASDR, age-standardized DALYs rate; DALYs, disability-adjusted life-years.

SUPPLEMENTARY FIGURE S3 The age distribution of lung cancer DALYs attributable to high fasting plasma glucose **(A)** and diet low in fruits **(B)** in different age groups, by sex, in 2021. The bars display the number of lung cancer DALYs. The lines represent ASDR. EAPC of global mortality rates of lung cancer attributable to high fasting plasma glucose in different age groups from 1990 to 2021, by sex **(C)** and SDI region **(E)**. EAPC of global mortality rates of lung cancer attributable to diet low in fruits in different age groups from 1990 to 2021, by sex **(D)** and SDI region **(F)**. ASDR, age-standardized DALYs rate; DALYs, disability-adjusted life-years; EAPC, estimated annual percentage change; CI, certainty interval; SDI, sociodemographic index.

SUPPLEMENTARY FIGURE S4 Correlations between ASDR of lung cancer attributable to high fasting plasma glucose and SDI at the regional **(A)** and national **(B)** level. Correlations between ASDR of lung cancer attributable to diet low in fruits and SDI at the regional **(C)** and national **(D)** level. The black line represents the expected ASDR on the basis of SDIs in all locations. ASDR, age-standardized DALYs rate; DALYs, disability-adjusted life-years; SDI, sociodemographic index.

SUPPLEMENTARY FIGURE S5 The actual and predicted values in ASDR of lung cancer attributable to high fasting plasma glucose **(A)** and diet low in fruits **(B)** with the ARIMA model by sex. ASDR, age-standardized DALYs rate; DALYs, disability-adjusted life-years; ARIMA, autoregressive integrated moving average.

SUPPLEMENTARY FIGURE S6 The actual and predicted values in ASDR of lung cancer attributable to high fasting plasma glucose **(A)** and diet low in fruits **(B)** with the ARIMA model by global and SDI regions. ASDR, age-standardized DALYs rate; DALYs, disability-adjusted life-years; SDI, sociodemographic index.

## Glossary

ACF1: Autocorrelation function at lag 1
AIC: Akaike information criterion
AICc: Corrected akaike information criterion
ARIMA: Autoregressive integrated moving average
ASR: Age-standardized Rate
ASDR: Age-Standardized DALYs Rate
ASMR: Age-Standardized Mortality Rate
BIC: Bayesian information criterion
CI: Confidence Interval
DALYs: Disability-Adjusted Life Years
DLF: Diet low in fruits
DM: Diabetes Mellitus
EAPC: Estimated Annual Percentage Change
GBD: Global Burden of Disease
HFPG: High fasting plasma glucose
LC: Lung Cancer
SCLC: Small Cell Lung Cancer
SDI: Socio-Demographic Index
NSCLC: Non-Small Cell Lung Cancer
UI: Uncertainty Interval
RMSE: Root mean square error
