# Supplementary figures and images for: Global burden of lung cancer attributable to metabolic and dietary risk factors: an overview of 3 decades and forecasted trends to 2036
Source: Front Nutr. 2025 Mar 13;12:1534106. doi: 10.3389/fnut.2025.1534106 (PMC11966415; doi:10.3389/fnut.2025.1534106)

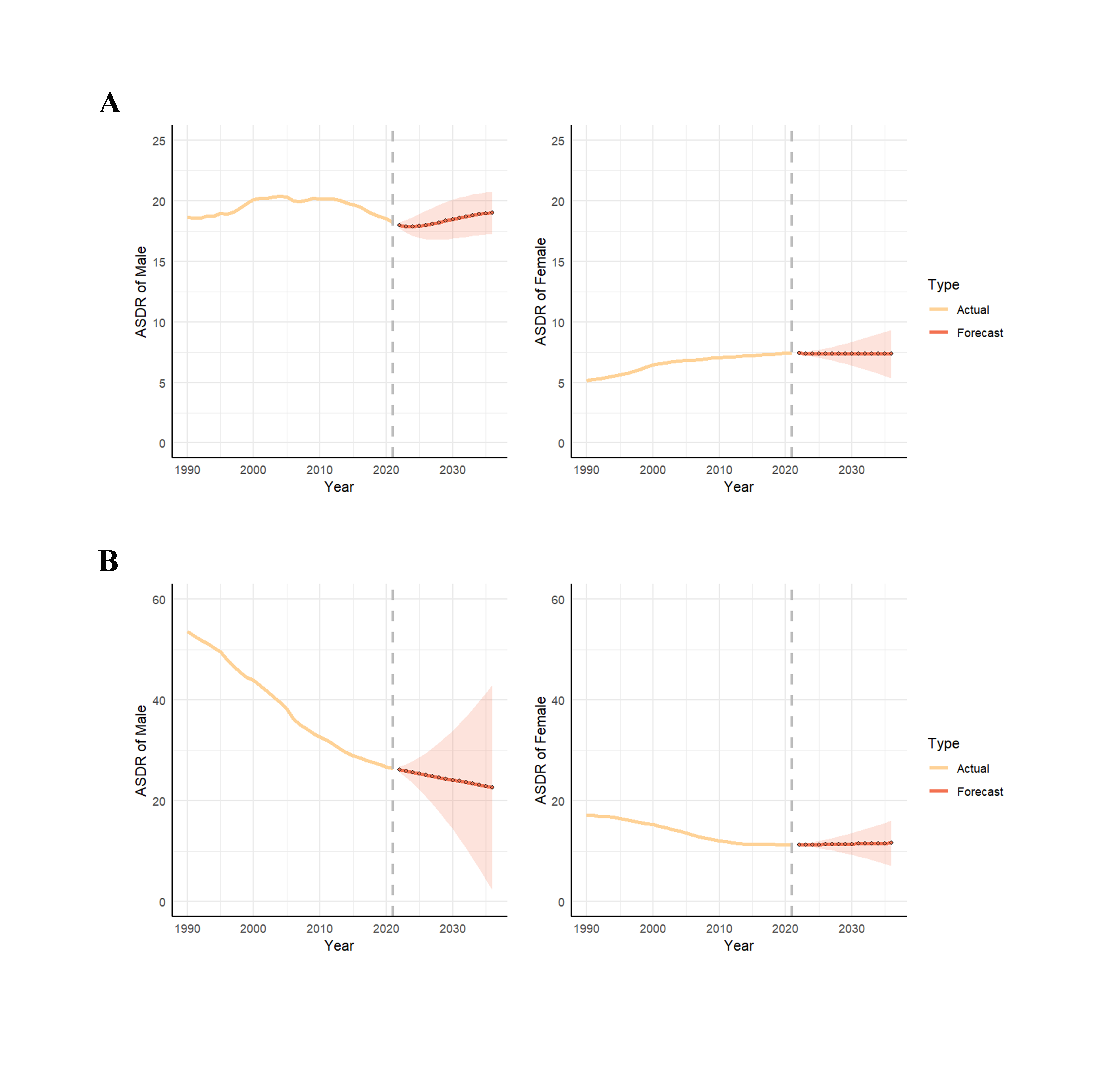

Supplement: SUPPLEMENTARY FIGURE S5 — The actual and predicted values in ASDR of lung cancer attributable to high fasting plasma glucose (A) and diet low in fruits (B) with the ARIMA model by sex. ASDR, age-standardized DALYs rate; DALYs, disability-adjusted life-years; ARIMA, autoregressive integrated moving average. [file Image_5.tif]
