# Supplementary material for: Global burden of lung cancer attributable to metabolic and dietary risk factors: an overview of 3 decades and forecasted trends to 2036
Source: Front Nutr. 2025 Mar 13;12:1534106. doi: 10.3389/fnut.2025.1534106 (PMC11966415; doi:10.3389/fnut.2025.1534106)
Supplement: Supplementary file 7 [file Table_1.docx]

# Supplementary Table S1. Regional deaths and ASMR of lung cancer attributable to high fasting plasma glucose in 1990 and 2021, and EAPC of ASMR from 1990 to 2021

| **Region** | **1990** | | **2021** | | **1990-2021** |
| --- | --- | --- | --- | --- | --- |
|  | **Number of Deaths (95%UI)** | **ASMR per 100,000 (95%UI)** | **Number of Deaths (95%UI)** | **ASMR per 100,000 (95%UI)** | **EAPC of ASMR (95%CI)** |
| Andean Latin America | 34.31(-7.08 to 76.65) | 0.18(-0.04 to 0.41) | 146.14(-27.54 to 342.89) | 0.26(-0.05 to 0.60) | 1.16 (1.02 to 1.30) |
| Australasia | 150.46(-30.80 to 331.22) | 0.62(-0.13 to 1.37) | 327.79(-65.03 to 712.36) | 0.58(-0.12 to 1.26) | -0.16 (-0.23 to -0.10) |
| Caribbean | 132.33(-26.76 to 294.91) | 0.53(-0.11 to 1.17) | 316.43(-62.85 to 741.43) | 0.59(-0.12 to 1.37) | 0.66 (0.54 to 0.78) |
| Central Asia | 136.14(-26.28 to 308.18) | 0.29(-0.06 to 0.64) | 219.49(-44.34 to 499.11) | 0.27(-0.06 to 0.62) | 0.42 (0.21 to 0.63) |
| Central Europe | 1,108.62(-225.87 to 2,474.53) | 0.72(-0.15 to 1.61) | 2,494.19(-511.01 to 5,652.52) | 1.08(-0.22 to 2.46) | 1.36 (1.24 to 1.49) |
| Central Latin America | 295.49(-60.90 to 669.10) | 0.39(-0.08 to 0.88) | 722.48(-146.54 to 1,654.21) | 0.30(-0.06 to 0.68) | -1.22 (-1.32 to -1.13) |
| Central Sub-Saharan Africa | 43.77(-8.33 to 105.91) | 0.22(-0.04 to 0.53) | 119.33(-19.92 to 287.51) | 0.24(-0.04 to 0.58) | 0.39 (0.11 to 0.66) |
| East Asia | 4,899.81(-1,053.78 to 11,209.80) | 0.61(-0.13 to 1.39) | 17,895.79(-3,560.59 to 41,043.39) | 0.82(-0.16 to 1.89) | 1.28 (1.00 to 1.57) |
| Eastern Europe | 1,078.51(-206.06 to 2,415.23) | 0.37(-0.07 to 0.83) | 1,317.08(-269.23 to 2,963.16) | 0.36(-0.07 to 0.81) | -0.08 (-0.21 to 0.04) |
| Eastern Sub-Saharan Africa | 50.15(-10.04 to 111.94) | 0.08(-0.02 to 0.17) | 138.88(-25.65 to 327.19) | 0.10(-0.02 to 0.23) | 0.70 (0.56 to 0.85) |
| High-income Asia Pacific | 1,240.41(-256.57 to 2,708.77) | 0.63(-0.13 to 1.38) | 3,505.98(-717.26 to 7,712.51) | 0.63(-0.13 to 1.39) | -0.19 (-0.38 to -0.01) |
| High-income North America | 3,897.65(-811.42 to 8,496.80) | 1.09(-0.23 to 2.38) | 7,475.73(-1520.14 to 1,6751.35) | 1.08(-0.22 to 2.40) | -0.06 (-0.39 to 0.26) |
| North Africa and Middle East | 526.60(-111.70 to 1,131.37) | 0.34(-0.07 to 0.73) | 2,331.54(-441.56 to 5,326.32) | 0.56(-0.11 to 1.28) | 1.93 (1.80 to 2.06) |
| Oceania | 12.27(-2.32 to 28.11) | 0.49(-0.09 to 1.11) | 38.34(-7.47 to 89.56) | 0.60(-0.12 to 1.39) | 0.68 (0.64 to 0.72) |
| South Asia | 646.53(-130.83 to 1,423.76) | 0.12(-0.02 to 0.26) | 2,705.72(-515.60 to 6,221.16) | 0.19(-0.04 to 0.43) | 1.32 (1.25 to 1.40) |
| Southeast Asia | 696.87(-142.86 to 1576.59) | 0.31(-0.06 to 0.69) | 2,874.03(-566.99 to 6,715.18) | 0.48(-0.09 to 1.12) | 1.19 (1.08 to 1.30) |
| Southern Latin America | 212.48(-43.28 to 466.40) | 0.46(-0.09 to 1.00) | 477.04(-98.39 to 1,077.78) | 0.53(-0.11 to 1.21) | 0.70 (0.57 to 0.83) |
| Southern Sub-Saharan Africa | 61.30(-11.39 to 144.42) | 0.25(-0.05 to 0.58) | 229.47(-43.30 to 539.99) | 0.43(-0.08 to 1.00) | 2.03 (1.70 to 2.36) |
| Tropical Latin America | 280.80(-53.61 to 637.29) | 0.32(-0.06 to 0.74) | 1,035.50(-205.65 to 2,329.54) | 0.41(-0.08 to 0.91) | 0.96 (0.88 to 1.04) |
| Western Europe | 3,433.11(-677.78 to 7,733.50) | 0.58(-0.11 to 1.30) | 6,018.11(-1,175.51 to 13,496.32) | 0.62(-0.12 to 1.38) | 0.33 (0.24 to 0.43) |
| Western Sub-Saharan Africa | 39.00(-7.58 to 85.33) | 0.05(-0.01 to 0.11) | 157.87(-27.74 to 366.15) | 0.09(-0.02 to 0.22) | 2.38 (2.27 to 2.50) |

Abbreviations: ASMR, age-standardized mortality rate; CI, confidential interval; EAPC, estimated annual percentage change; UI, uncertainty interval.
