# Supplementary material for: Global burden of lung cancer attributable to metabolic and dietary risk factors: an overview of 3 decades and forecasted trends to 2036
Source: Front Nutr. 2025 Mar 13;12:1534106. doi: 10.3389/fnut.2025.1534106 (PMC11966415; doi:10.3389/fnut.2025.1534106)
Supplement: Supplementary file 8 [file Table_2.docx]

# Supplementary Table S2. Regional DALYs and ASDR of lung cancer attributable to high fasting plasma glucose in 1990 and 2021, and EAPC of ASDR from 1990 to 2021

| **Region** | **1990** | | **2021** | | **1990-2021** |
| --- | --- | --- | --- | --- | --- |
|  | **DALYs (95%UI)** | **ASDR per 100,000 (95%UI)** | **DALYs (95%UI)** | **ASDR per 100,000 (95%UI)** | **EAPC of ASDR (95%CI)** |
| Andean Latin America | 781.80(-160.86 to 1,733.71) | 3.91(-0.80 to 8.71) | 3,029.56(-575.12 to 7,157.45) | 5.19(-0.98 to 12.26) | 0.90 (0.74 to 1.06) |
| Australasia | 3,388.41(-700.99 to 7,409.11) | 14.26(-2.96 to 31.16) | 6,529.59(-1,305.93 to 14,131.12) | 12.33(-2.46 to 26.76) | -0.43 (-0.48 to -0.38) |
| Caribbean | 2,860.13(-585.19 to 6,336.92) | 11.05(-2.26 to 24.48) | 6,858.05(-1,375.07 to 16,083.33) | 12.69(-2.54 to 29.78) | 0.71 (0.61 to 0.81) |
| Central Asia | 3,815.68(-731.75 to 8,602.83) | 7.67(-1.48 to 17.30) | 5,671.83(-1,152.25 to 12,987.15) | 6.57(-1.33 to 15.03) | -0.05 (-0.23 to 0.13) |
| Central Europe | 28,498.92(-5,824.42 to 63,126.03) | 18.33(-3.75 to 40.66) | 55,274.29(-11,392.78 to 125,239.59) | 24.98(-5.16 to 56.71) | 1.05 (0.90 to 1.19) |
| Central Latin America | 6,894.91(-1,425.81 to 15,416.70) | 8.43(-1.74 to 18.96) | 15,981.65(-3,261.52 to 36,494.40) | 6.37(-1.30 to 14.56) | -1.29 (-1.39 to -1.19) |
| Central Sub-Saharan Africa | 1,171.25(-222.55 to 2,846.48) | 5.05(-0.96 to 12.26) | 3,218.44(-541.39 to 7,775.00) | 5.67(-0.95 to 13.67) | 0.39 (0.12 to 0.66) |
| East Asia | 12,4850.00(-26,716.17 to 28,4842.58) | 13.96(-3.00 to 31.81) | 397,025.63(-79,119.77 to 910,568.85) | 17.59(-3.52 to 40.38) | 1.05 (0.80 to 1.30) |
| Eastern Europe | 28,869.48(-5,491.20 to 64,734.88) | 9.87(-1.88 to 22.20) | 31,498.21(-6,457.56 to 70,969.58) | 8.77(-1.80 to 19.83) | -0.46 (-0.59 to -0.33) |
| Eastern Sub-Saharan Africa | 1,255.72(-250.28 to 2,779.26) | 1.70(-0.34 to 3.78) | 3,294.47(-608.97 to 7,821.74) | 2.06(-0.38 to 4.85) | 0.45 (0.28 to 0.62) |
| High-income Asia Pacific | 26,136.12(-5,420.97 to 56,780.42) | 12.86(-2.66 to 27.88) | 56,157.71(-11,530.77 to 124,103.14) | 11.51(-2.36 to 25.67) | -0.55 (-0.75 to -0.36) |
| High-income North America | 89,301.30(-18,489.96 to 193,896.50) | 26.11(-5.4 to 57.04) | 148,484.32(-30,415.87 to 327,806.49) | 22.10(-4.54 to 48.66) | -0.58 (-0.88 to -0.28) |
| North Africa and Middle East | 13,361.78(-2,881.08 to 28,779.03) | 7.79(-1.66 to 16.72) | 56,060.35(-10,641.33 to 127,922.92) | 12.31(-2.33 to 28.04) | 1.72 (1.59 to 1.84) |
| Oceania | 316.36(-60.20 to 731.16) | 10.89(-2.05 to 24.87) | 975.70(-189.37 to 2,291.31) | 13.23(-2.58 to 30.93) | 0.65 (0.62 to 0.69) |
| South Asia | 17,337.37(-3,496.22 to 37,956.23) | 2.89(-0.58 to 6.35) | 68,784.17(-13,356.53 to 157,124.63) | 4.49(-0.87 to 10.29) | 1.30 (1.23 to 1.36) |
| Southeast Asia | 16,768.72(-3,427.53 to 38,356.63) | 6.72(-1.38 to 15.29) | 66,117.42(-13,186.75 to 155,062.39) | 10.10(-2.01 to 23.65) | 1.05 (0.96 to 1.15) |
| Southern Latin America | 5,153.71(-1,053.65 to 11,314.97) | 10.92(-2.24 to 24.02) | 10,261.52(-2,135.50 to 23,093.85) | 11.75(-2.45 to 26.36) | 0.39 (0.25 to 0.53) |
| Southern Sub-Saharan Africa | 1,479.35(-276.41 to 3,502.60) | 5.49(-1.02 to 13.02) | 5,576.03(-1,056.29 to 13,080.86) | 9.54(-1.80 to 22.42) | 2.05 (1.71 to 2.40) |
| Tropical Latin America | 6,936.69(-1,333.98 to 15,570.26) | 7.48(-1.44 to 16.85) | 23,528.12(-4,636.37 to 52,643.91) | 9.04(-1.78 to 20.26) | 0.80 (0.72 to 0.88) |
| Western Europe | 75,584.54(-14,999.76 to 170,636.41) | 13.25(-2.64 to 29.95) | 116,980.97(-22,915.81 to 262,421.91) | 13.13(-2.58 to 29.53) | 0.08 (-0.02 to 0.18) |
| Western Sub-Saharan Africa | 917.11(-178.44 to 2,005.18) | 1.07(-0.21 to 2.33) | 3,714.91(-657.51 to 8,539.43) | 1.98(-0.35 to 4.61) | 2.30 (2.20 to 2.41) |

Abbreviations: ASDR, age-standardized DALYs rate; CI, confidential interval; DALY, disability-adjusted life-year; EAPC, estimated annual percentage change; UI, uncertainty interval.
