# Supplementary material for: Global burden of lung cancer attributable to metabolic and dietary risk factors: an overview of 3 decades and forecasted trends to 2036
Source: Front Nutr. 2025 Mar 13;12:1534106. doi: 10.3389/fnut.2025.1534106 (PMC11966415; doi:10.3389/fnut.2025.1534106)
Supplement: Supplementary file 9 [file Table_3.docx]

| **Region** | **1990** | | **2021** | | **1990-2021** |
| --- | --- | --- | --- | --- | --- |
|  | **Number of Deaths (95%UI)** | **ASMR per 100,000 (95%UI)** | **Number of Deaths (95%UI)** | **ASMR per 100,000 (95%UI)** | **EAPC of ASMR (95%CI)** |
| Andean Latin America | 78.43(39.65 to 122.29) | 0.40(0.20 to 0.62) | 128.37(61.86 to 200.19) | 0.22(0.11 to 0.34) | -2.10(-2.35 to -1.85) |
| Australasia | 240.12(119.86 to 350.58) | 1.01(0.50 to 1.47) | 328.70(168.98 to 487.59) | 0.59(0.31 to 0.88) | -1.71 (-1.76 to -1.66) |
| Caribbean | 124.28(61.78 to 185.51) | 0.49(0.24 to 0.72) | 167.05(80.68 to 247.90) | 0.31(0.15 to 0.46) | -1.42 (-1.52 to -1.33) |
| Central Asia | 859.60(438.46 to 1,252.09) | 1.78(0.90 to 2.59) | 381.08(196.46 to 559.65) | 0.46(0.24 to 0.68) | -4.75 (-4.96 to -4.54) |
| Central Europe | 2,074.49(1,054.19 to 3,002.17) | 1.37(0.70 to 1.99) | 2,223.53(1,135.99 to 3,283.08) | 1.00(0.51 to 1.47) | -1.20 (-1.41 to -0.99) |
| Central Latin America | 252.14(126.81 to 366.41) | 0.32(0.16 to 0.46) | 483.46(243.80 to 710.97) | 0.20(0.10 to 0.29) | -1.71 (-1.79 to -1.64) |
| Central Sub-Saharan Africa | 86.46(39.29 to 152.22) | 0.39(0.18 to 0.68) | 251.66(100.60 to 466.00) | 0.46(0.19 to 0.84) | 0.43 (0.30 to 0.56) |
| East Asia | 17,899.99(8,752.23 to 27,450.77) | 2.12(1.04 to 3.22) | 19,553.39(9,988.82 to 30,556.80) | 0.92(0.47 to 1.44) | -2.99 (-3.16 to -2.82) |
| Eastern Europe | 5,642.04(2,927.66 to 8,218.47) | 1.97(1.02 to 2.87) | 2,825.95(1,449.49 to 4,147.27) | 0.80(0.41 to 1.17) | -3.60 (-3.86 to -3.33) |
| Eastern Sub-Saharan Africa | 692.86(363.25 to 1,065.38) | 0.91(0.48 to 1.40) | 1025.18(522.30 to 1,468.28) | 0.65(0.33 to 0.93) | -1.30 (-1.44 to -1.17) |
| High-income Asia Pacific | 2,045.27(1,054.21 to 2,952.98) | 1.04(0.54 to 1.49) | 3,788.85(1,927.38 to 5,598.52) | 0.72(0.36 to 1.06) | -1.00 (-1.19 to -0.80) |
| High-income North America | 4,797.84(2,456.91 to 7,008.65) | 1.37(0.70 to 2.01) | 4,753.87(2,371.77 to 7,308.42) | 0.70(0.35 to 1.07) | -2.13 (-2.21 to -2.06) |
| North Africa and Middle East | 464.78(228.43 to 726.32) | 0.28(0.14 to 0.44) | 844.81(424.82 to 1,277.11) | 0.19(0.10 to 0.29) | -1.44 (-1.54 to -1.35) |
| Oceania | 26.13(11.91 to 44.32) | 0.95(0.44 to 1.63) | 57.02(25.93 to 96.17) | 0.82(0.38 to 1.36) | -0.41 (-0.48 to -0.34) |
| South Asia | 6,186.05(3,329.67 to 9,062.93) | 1.06(0.57 to 1.56) | 16,739.22(8,630.71 to 23,927.20) | 1.12(0.58 to 1.61) | 0.10 (0.02 to 0.17) |
| Southeast Asia | 3,462.44(1,761.77 to 5,213.15) | 1.38(0.7 to 2.07) | 4,374.16(2,127.22 to 6,611.79) | 0.68(0.33 to 1.03) | -2.53 (-2.65 to -2.41) |
| Southern Latin America | 326.23(164.36 to 475.61) | 0.70(0.35 to 1.03) | 279.92(142.19 to 421.19) | 0.32(0.16 to 0.48) | -2.55 (-2.68 to -2.41) |
| Southern Sub-Saharan Africa | 462.63(242.81 to 701.08) | 1.70(0.90 to 2.58) | 1,067.82(542.64 to 1,556.32) | 1.86(0.95 to 2.68) | 0.20 (-0.15 to 0.56) |
| Tropical Latin America | 322.21(162.97 to 475.26) | 0.36(0.18 to 0.54) | 576.37(296.17 to 858.95) | 0.23(0.12 to 0.34) | -1.63 (-1.71 to -1.54) |
| Western Europe | 5,340.42(2,698.58 to 7,762.14) | 0.92(0.47 to 1.34) | 5,650.38(2,848.59 to 8,259.26) | 0.60(0.30 to 0.87) | -1.21 (-1.30 to -1.12) |
| Western Sub-Saharan Africa | 236.96(119.49 to 352.28) | 0.28(0.14 to 0.42) | 544.65(267.32 to 815.09) | 0.30(0.15 to 0.45) | 0.45 (0.35 to 0.55) |

# Supplementary Table S3. Regional deaths and ASMR of lung cancer attributable to diet low in fruits in 1990 and 2021, and EAPC of ASMR from 1990 to 2021

Abbreviations: ASMR, age-standardized mortality rate; CI, confidential interval; EAPC, estimated annual percentage change; UI, uncertainty interval.
