# Supplementary material for: Global burden of lung cancer attributable to metabolic and dietary risk factors: an overview of 3 decades and forecasted trends to 2036
Source: Front Nutr. 2025 Mar 13;12:1534106. doi: 10.3389/fnut.2025.1534106 (PMC11966415; doi:10.3389/fnut.2025.1534106)
Supplement: Supplementary file 10 [file Table_4.docx]

| **Region** | **1990** | | **2021** | | **1990-2021** |
| --- | --- | --- | --- | --- | --- |
|  | **DALYs (95%UI)** | **ASDR per 100,000 (95%UI)** | **DALYs (95%UI)** | **ASDR per 100,000 (95%UI)** | **EAPC of ASDR (95%CI)** |
| Andean Latin America | 2,084.74(1,060.79 to 3,221.80) | 9.59(4.86 to 14.87) | 3,053.10(1,460.15 to 4,756.15) | 5.06(2.42 to 7.86) | -2.33 (-2.62 to -2.03) |
| Australasia | 5,697.55(2,861.23 to 8,319.46) | 24.37(12.22 to 35.55) | 6,699.61(3,486.33 to 9,909.47) | 13.06(6.83 to 19.32) | -2.00 (-2.07 to -1.94) |
| Caribbean | 3,071.37(1,528.78 to 4,605.88) | 11.61(5.77 to 17.42) | 3,953.66(1,895.85 to 5,867.19) | 7.35(3.52 to 10.89) | -1.51 (-1.60 to -1.41) |
| Central Asia | 26,223.16(13,391.42 to 38,103.33) | 52.05(26.47 to 75.81) | 10,853.60(5,571.65 to 15,980.34) | 12.13(6.24 to 17.81) | -5.16 (-5.38 to -4.93) |
| Central Europe | 58,378.24(29,834.89 to 84,084.65) | 38.55(19.69 to 55.54) | 51,738.42(26,282.77 to 76,270.88) | 24.62(12.50 to 36.26) | -1.63 (-1.86 to -1.40) |
| Central Latin America | 6,687.26(3,362.16 to 9,669.58) | 7.56(3.80 to 10.97) | 11,772.24(5,938.96 to 17,378.16) | 4.62(2.33 to 6.82) | -1.77 (-1.85 to -1.70) |
| Central Sub-Saharan Africa | 2,584.18(1,183.38 to 4,532.75) | 10.18(4.63 to 17.96) | 7,603.64(3,019.61 to 14,241.55) | 11.94(4.78 to 22.04) | 0.38 (0.24 to 0.51) |
| East Asia | 520,775.78(255,283.07 to 780,491.09) | 54.78(26.76 to 82.71) | 455,283.76(229,549.26 to 716,373.34) | 20.72(10.48 to 32.5) | -3.49 (-3.65 to -3.33) |
| Eastern Europe | 164,166.26(85,551.40 to 238,346.15) | 57.59(30.06 to 83.50) | 72,401.66(37,070.44 to 105,901.21) | 21.24(10.90 to 31.11) | -3.96 (-4.25 to -3.68) |
| Eastern Sub-Saharan Africa | 20,325.02(10,574.70 to 31,505.51) | 24.45(12.77 to 37.66) | 28,946.99(14,805.10 to 41,514.4) | 15.65(7.97 to 22.36) | -1.70 (-1.85 to -1.56) |
| High-income Asia Pacific | 47,430.45(24,351.48 to 68,656.75) | 23.25(11.95 to 33.66) | 65,117.03(32,978.25 to 96,289.81) | 15.02(7.65 to 22.08) | -1.15 (-1.38 to -0.91) |
| High-income North America | 116,402.99(59,213.30 to 170,119.53) | 34.99(17.78 to 50.97) | 98,586.44(49,205.03 to 150,589.69) | 15.31(7.67 to 23.30) | -2.62 (-2.69 to -2.56) |
| North Africa and Middle East | 13,430.08(6,595.65 to 21,037.66) | 7.25(3.56 to 11.35) | 23,505.73(11,719.35 to 35,334.63) | 4.70(2.36 to 7.10) | -1.62 (-1.71 to -1.53) |
| Oceania | 762.29(346.10 to 1,300.83) | 23.56(10.70 to 40.04) | 1,643.02(735.44 to 2,810.26) | 19.92(9.07 to 33.59) | -0.46 (-0.53 to -0.38) |
| South Asia | 185,148.31(99,645.07 to 270,387.56) | 28.61(15.39 to 41.85) | 469,725.09(241,738.98 to 673,435.24) | 29.62(15.24 to 42.45) | 0.03 (-0.05 to 0.10) |
| Southeast Asia | 99,019.98(50,496.87 to 149,303.27) | 35.60(18.14 to 53.73) | 118,213.87(57,829.34 to 179,100.18) | 16.87(8.24 to 25.48) | -2.67 (-2.80 to -2.54) |
| Southern Latin America | 8,790.76(4,446.48 to 12,844.04) | 18.75(9.49 to 27.41) | 6,388.76(3,226.03 to 9,596.85) | 7.47(3.75 to 11.23) | -3.00 (-3.13 to -2.86) |
| Southern Sub-Saharan Africa | 13,674.30(7,150.69 to 20,679.83) | 46.05(24.07 to 69.57) | 30,227.94(15,316.89 to 43,759.08) | 48.06(24.4 to 69.84) | 0.06 (-0.31 to 0.42) |
| Tropical Latin America | 8,993.12(4,514.61 to 13,360.05) | 9.14(4.60 to 13.56) | 13,945.02(7,176.54 to 20,904.29) | 5.34(2.76 to 8.01) | -1.93 (-2.02 to -1.85) |
| Western Europe | 125,376.02(63,353.47 to 181,484.93) | 22.91(11.58 to 33.11) | 116,950.48(59,350.07 to 169,417.31) | 13.97(7.06 to 20.24) | -1.42 (-1.49 to -1.34) |
| Western Sub-Saharan Africa | 6,353.30(3,193.60 to 9,375.72) | 6.85(3.45 to 10.14) | 14,656.99(7,088.00 to 22,043.05) | 7.01(3.43 to 10.46) | 0.30 (0.20 to 0.41) |

# Supplementary Table S4. Regional DALYs and ASDR of lung cancer attributable to diet low in fruits in 1990 and 2021, and EAPC of ASDR from 1990 to 2021

Abbreviations: ASDR, age-standardized DALYs rate; CI, confidential interval; DALY, disability-adjusted life-year; EAPC, estimated annual percentage change; UI, uncertainty interval.
