# Supplementary material for: Global burden of lung cancer attributable to metabolic and dietary risk factors: an overview of 3 decades and forecasted trends to 2036
Source: Front Nutr. 2025 Mar 13;12:1534106. doi: 10.3389/fnut.2025.1534106 (PMC11966415; doi:10.3389/fnut.2025.1534106)
Supplement: Supplementary file 11 [file Table_5.docx]

| **Characteristics** | **ASMR** | | | | | | **ASDR** | | | | | |
| --- | --- | --- | --- | --- | --- | --- | --- | --- | --- | --- | --- | --- |
|  | **(p, q, d)** | **AIC** | **AICc** | **BIC** | **RMSE** | **ACF1** | **(p, q, d)** | **AIC** | **AICc** | **BIC** | **RMSE** | **ACF1** |
| **High fasting plasma glucose** | (0,2,0) | -244.51 | -244.37 | -243.11 | 0.003851806 | -0.14079160 | (0,2,0) | -63.88 | -63.73 | -62.47 | 0.07820039 | -0.14797420 |
| **Sex** |  |  |  |  |  |  |  |  |  |  |  |  |
| Male | (0,2,0) | -211.14 | -211 | -209.74 | 0.006717267 | -0.11084780 | (2,0,0) | -28.36 | -26.87 | -22.49 | 0.12767960 | -0.01236200 |
| Female | (0,2,1) | -284.29 | -283.85 | -281.49 | 0.001913423 | -0.05072218 | (1,2,0) | -107.27 | -106.83 | -104.47 | 0.03660923 | -0.03378105 |
| **SDI region** |  |  |  |  |  |  |  |  |  |  |  |  |
| High SDI | (2,2,2) | -209.64 | -207.14 | -202.64 | 0.005796925 | -0.04494961 | (1,2,0) | -23.62 | -23.18 | -20.82 | 0.14701870 | -0.08097925 |
| High-middle SDI | (0,2,0) | -201.55 | -201.41 | -200.15 | 0.007878950 | -0.03638536 | (0,2,0) | -19.82 | -19.68 | -18.42 | 0.16288640 | -0.04835791 |
| Middle SDI | (0,1,1) | -231.42 | -230.53 | -227.12 | 0.005079394 | 0.07750859 | (1,1,0) | -47.41 | -46.98 | -44.54 | 0.10290800 | 0.10196930 |
| Low-middle SDI | (1,1,0) | -332.14 | -331.25 | -327.83 | 0.001017027 | -0.02032652 | (1,1,0) | -139.70 | -138.81 | -135.40 | 0.02263839 | -0.05015340 |
| Low SDI | (0,2,0) | -359.37 | -359.23 | -357.97 | 0.0005680869 | -0.21045660 | (0,2,1) | -176.38 | -175.94 | -173.58 | 0.01157546 | -0.04549028 |
| **Diet low in fruits** | (1,1,0) | -219.61 | -218.72 | -215.31 | 0.006213943 | -0.00266345 | (0,2,0) | -20.62 | -20.47 | -19.21 | 0.16095110 | -0.16820370 |
| **Sex** |  |  |  |  |  |  |  |  |  |  |  |  |
| Male | (1,1,0) | -185.29 | -184.4 | -180.99 | 0.010837010 | 0.01204615 | (0,2,0) | 13.97 | 14.11 | 15.37 | 0.28635400 | -0.21336310 |
| Female | (1,1,0) | -269.15 | -268.72 | -266.28 | 0.002833250 | -0.21302700 | (0,2,0) | -77.06 | -76.92 | -75.66 | 0.06287763 | -0.08599133 |
| **SDI region** |  |  |  |  |  |  |  |  |  |  |  |  |
| High SDI | (0,1,0) | -219.52 | -219.1 | -216.66 | 0.006477332 | -0.14211860 | (1,1,0) | -219.61 | -218.72 | -215.31 | 0.00621394 | -0.00266345 |
| High-middle SDI | (1,1,0) | -166.52 | -165.63 | -162.22 | 0.014613740 | 0.12625230 | (1,1,0) | 38.97 | 39.86 | 43.27 | 0.40141220 | 0.14211770 |
| Middle SDI | (0,2,0) | -186.10 | -185.96 | -184.70 | 0.010197380 | -0.03915293 | (0,2,0) | -6.98 | -6.83 | -5.58 | 0.20196270 | -0.00999441 |
| Low-middle SDI | (0,2,2) | -203.69 | -202.77 | -199.49 | 0.007016732 | -0.07486025 | (0,2,0) | -14.14 | -14.00 | -12.74 | 0.17912970 | -0.18230060 |
| Low SDI | (0,1,1) | -248.64 | -248.22 | -245.78 | 0.004005058 | -0.02266932 | (0,2,3) | -53.03 | -51.43 | -47.43 | 0.08382320 | -0.02348700 |

# Supplementary Table S5. The parameters of ARIMA model to predict ASMR and ASDR for lung cancer from 2021 to 2036

Abbreviations: ARIMA, autoregressive integrated moving average; ASMR, age-standardized mortality rate; ASDR, age-standardized DALYs rate; DALY, disability-adjusted life-year; AIC, akaike information criterion; AICc: corrected akaike information criterion; BIC, bayesian information criterion; RMSE: root mean square error; ACF1: autocorrelation function at lag 1.
